# Key Features of Human Episodic Recollection in the Cross-Episode Retrieval of Rat Hippocampus Representations of Space

**DOI:** 10.1371/journal.pbio.1001607

**Published:** 2013-07-16

**Authors:** Eduard Kelemen, André A. Fenton

**Affiliations:** 1 Department of Physiology and Pharmacology, State University of New York, Downstate Medical Center, Brooklyn, New York, United States of America; 2 The Robert F. Furchgott Center for Neural and Behavioral Science, State University of New York, Downstate Medical Center, Brooklyn, New York, United States of America; 3 Center for Neural Science, New York University, New York, New York, United States of America; Boston University, United States of America

## Abstract

Neurophysiological studies focus on memory retrieval as a reproduction of what was experienced and have established that neural discharge is replayed to express memory. However, cognitive psychology has established that recollection is not a verbatim replay of stored information. Recollection is constructive, the product of memory retrieval cues, the information stored in memory, and the subject's state of mind. We discovered key features of constructive recollection embedded in the rat CA1 ensemble discharge during an active avoidance task. Rats learned two task variants, one with the arena stable, the other with it rotating; each variant defined a distinct behavioral episode. During the rotating episode, the ensemble discharge of CA1 principal neurons was dynamically organized to concurrently represent space in two distinct codes. The code for spatial reference frame switched rapidly between representing the rat's current location in either the stationary spatial frame of the room or the rotating frame of the arena. The code for task variant switched less frequently between a representation of the current rotating episode and the stable episode from the rat's past. The characteristics and interplay of these two hippocampal codes revealed three key properties of constructive recollection. (1) Although the ensemble representations of the stable and rotating episodes were distinct, ensemble discharge during rotation occasionally resembled the stable condition, demonstrating cross-episode retrieval of the representation of the remote, stable episode. (2) This cross-episode retrieval at the level of the code for task variant was more likely when the rotating arena was about to match its orientation in the stable episode. (3) The likelihood of cross-episode retrieval was influenced by preretrieval information that was signaled at the level of the code for spatial reference frame. Thus key features of episodic recollection manifest in rat hippocampal representations of space.

## Abstract 2

Using a rat model of episodic memory, the authors show that recollection is not a verbatim replay of stored experience, but is rather an intermingling of stored memory, environmental circumstances, and the subject's state of mind.

## Author Summary

Since Plato and the early cognitive psychologists we have believed that recollection is not *a straightforward replay of stored experience*; memory can be modified by the subject's experience and or state of mind, sometimes generating knowledge that was never itself experienced. We show in rats that direct neurophysiological evidence that episodic recollection is not a verbatim replay of stored experience; rather retrieval is a constructive process—an intermingling of the stored memory, environmental circumstances, and the subject's state of mind. To monitor internal, neural representations, we recorded the action potential discharge within a network of hippocampal neurons while rats performed two familiar active place avoidance tasks: one where the arena was stable and one where it rotated. Neural representations of the stable and rotating conditions were distinct. However, during the rotating task we intermittently observed intervals that we call “cross-episode retrieval” because discharge resembled the representation of the stable task. The incidence of cross-episode retrieval was higher when the angular position of the rotating arena was similar to that in the stable condition, demonstrating that retrieval is influenced by the environment. Cross-episode retrieval was also more likely when discharge represented the position of the rat in the stationary room rather than when it represented positions that rotate with the arena; this demonstrated that retrieval is influenced by internal cognitive variables that are encoded by hippocampal discharge—a state of mind. Thus novel, key features of constructive human episodic memory can be observed in rat hippocampal discharge.

## Introduction

Cognitive psychologists have long understood that memory retrieval is not a straightforward reproduction of stored information but is rather a (re)constructive process that characteristically modifies the stored and recollected information [1]. The theory of constructive recollection asserts that information across different behavioral episodes may combine during recall to form what is recollected as a single experience. For example, people mistakenly recalled seeing a school bus in a movie, if the bus was mentioned after they watched the movie [2]. The theory of constructive recollection also asserts that the retrieval cues can modify the content of the retrieved information. For example, after seeing a film clip of a car accident, people are more likely to report remembering a broken headlight when asked “Did you see *the* broken headlight?” than when asked “Did you see *a* broken headlight?” [3]. Furthermore, a subject's current mental state or mindset can also influence the retrieved information. For example, looking at a house from the perspective of a homebuyer or a burglar leads to different recollections; in the former case features like the leaky roof are recalled, and in the latter case, the precious collection of coins [4]. On the basis of such observations, the constructive theory has established that explicit memory retrieval is the result of complex interactions between the retrieval cues, information stored from prior experiences, and the subject's state of mind. This process is known as “ecphoria” [5].

In contrast to the detailed knowledge of constructive memory retrieval provided by cognitive psychology, very little is known about the neural activity that underlies this process. However, the accumulating knowledge about the dynamical organization of hippocampal ensemble activity provides important clues. The subsequent reactivation of single cell discharge patterns from previous experiences has been identified as a candidate neuronal signature of memory retrieval [6] and properties of hippocampal reactivation have been characterized in detail under various experimental manipulations in rats [7],[8]. Furthermore, the dynamic nature of hippocampal ensemble activity has been recognized and characterized during steady state conditions [9]–[11] as well as during transitions between distinct representations [12]. Although this previous work did not attempt to explore the neural mechanism behind constructive memory retrieval, it has paved the way for a systematic attempt to bridge the gap between the psychologically characterized properties of memory retrieval and neuronal ensemble activity, its presumed neural substrate.

Investigating the neural substrate of constructive memory retrieval requires a hippocampus-dependent learning paradigm that permits studying the interaction between different memories, and associating neural activity with cognitive behavior. We have previously recorded hippocampal ensemble discharge while rats perform a shock-motivated active place avoidance task and demonstrated that dynamic patterns of ensemble discharge represent the information the rat is currently using to locate itself and shock punishment [13]. Here we study hippocampal ensemble discharge in rats performing two variants of the active place avoidance task to investigate the interactions between two distinct hippocampal mnemonic representations. This puts us in a unique position to investigate the hippocampal ensemble discharge that underlies constructive memory retrieval.

We investigated the neural activity correlates of recollection from the combined standpoint of the constructive theory and the established neurophysiologist view. Since recollections combine information from multiple episodes and recall is associated with the replay of neural activity, then recollection must involve cross-episode neuronal reactivation. Here we use the term “cross-episode retrieval” to mean that in one behavioral condition, neural activity occurs that is characteristic of a distinct condition that was experienced earlier. Such cross-episode expression of past activity can create opportunities for generating novel associations and new information that was never directly experienced. We examined ensemble discharge patterns of hippocampus CA1 principal neurons to evaluate three predictions of the constructive view: (1) discharge that is characteristic of the previously experienced condition and stored memory should co-express with the discharge that is characteristic of the current episode, demonstrating cross-episode retrieval; (2) external retrieval cues should influence the cross-episode expression of the memory-associated discharge; and (3) information in preretrieval hippocampal discharge should influence cross-episode retrieval of the memory-associated discharge.

## Results

**Figure 1:**
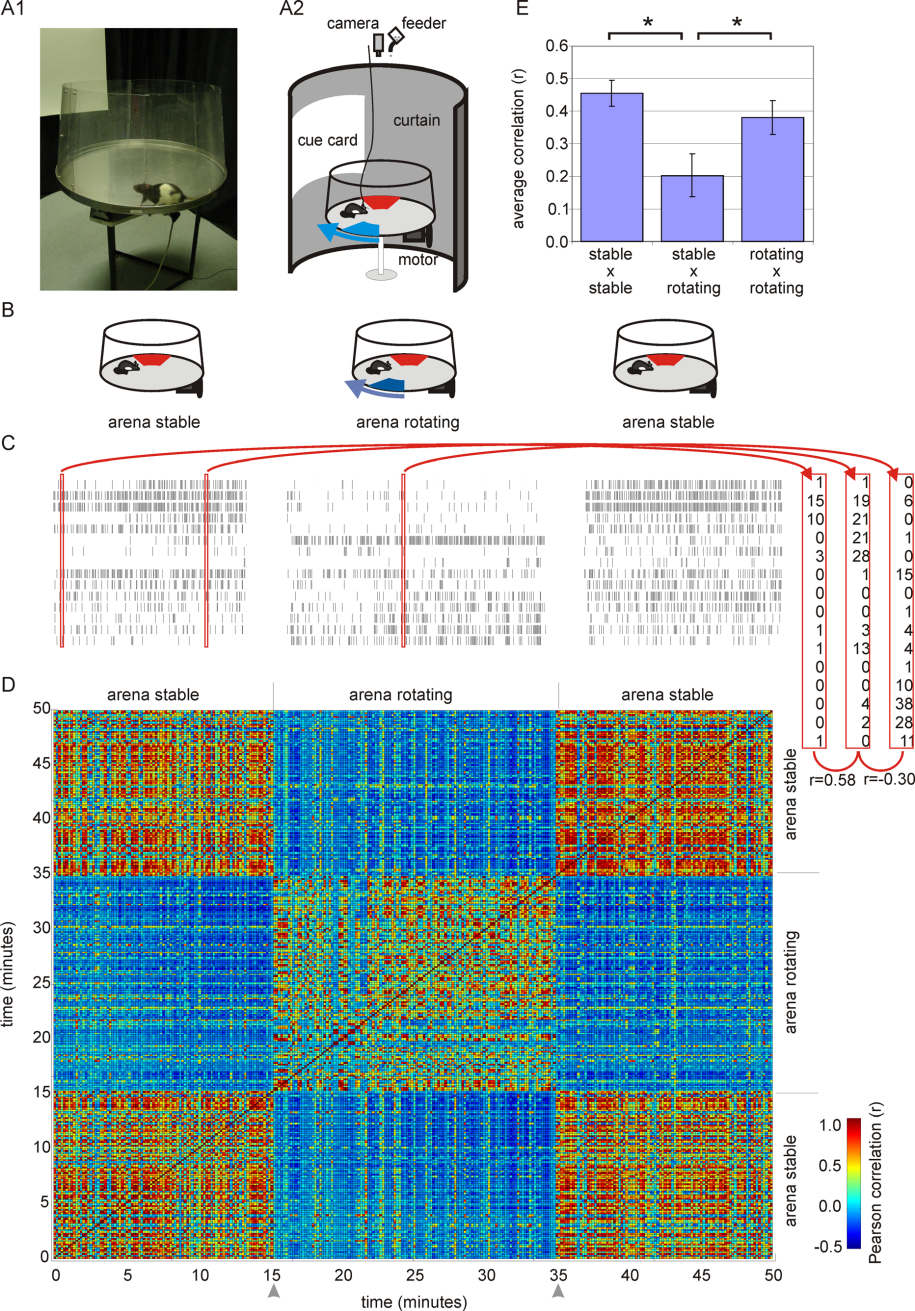
Hippocampal ensemble discharge during the stable and rotating conditions. (A) A photograph of a rat on the arena (A1) and a schematic drawing of the apparatus during the rotating condition (A2). A rat was placed on a circular arena that was surrounded by a black curtain with a white cue card. The arena rotated slowly in the rotating condition and was otherwise stable. The rat was reinforced to avoid two unmarked shock zones. A room frame shock zone (red) was defined relative to room landmarks and did not rotate. An arena frame shock zone (blue) was defined relative to arena landmarks and rotated together with the arena as indicated by the blue arrow. (B) Schematics of the experimental protocol. Each hippocampal ensemble was recorded during one session of rotating condition, flanked by two sessions of the stable condition. (C) Raster plots of activity of an ensemble of 15 cells during the stable and rotating conditions in the same environment. For each 10-s interval, the ensemble activity was characterized by a spike-count vector (red rectangles). The similarity of ensemble activity during any two intervals was assessed by computing the Pearson correlation between the corresponding ensemble vectors. (D) The correlation matrix shows that the correlation of ensemble activity for each pair of 10-s intervals recorded during the stable condition tends to be high. Similarly, the intervals recorded during the rotating condition tend to have highly correlated activity. Intervals during rotation were often dissimilar to the intervals during the stable condition, as is indicated by blue pixels. (E) Average of the correlation between ensemble activity during different intervals in the stable and rotating conditions (data from all recordings). Correlations are high when activity in the same conditions is compared and lower when activity is compared between the stationary and rotating conditions.

To test these three predictions of the constructive hypothesis, we studied hippocampus ensemble discharge during two distinct task variants of the active place avoidance paradigm (Figure 1A) [14] that resulted in correspondingly distinct hippocampus ensemble discharge patterns [13]. In one task, the behavioral arena was stable, and in the other it was rotating, allowing us to switch between the stable and rotating conditions by remotely starting or stopping the rotation. Each ensemble of hippocampus neurons was recorded in a sequence of stable–rotating–stable conditions (Figure 1B). During the rotating condition recordings, the environment was in an effective dynamic steady state and no perturbations were introduced. We began by searching for the neural signature of the stable condition while the rat was doing the avoidance task in the rotating condition.

### Change in Ensemble Activity Between Stationary and Rotating Conditions

First we characterized the reproducibility of ensemble activity across each 10-s interval of the stationary and rotating conditions. The stationary activity patterns were highly correlated, indicating self-similarity (r = 0.46±0.04; Figure 1D). Ensemble activity during the rotating condition was also self-similar (r = 0.38±0.05), but activity in the stable and rotating conditions was more distinctive (r = 0.20±0.07), suggesting that ensemble activity changed to represent the two different conditions (Figure 1E). Indeed, the similarity of ensemble activity from the stable and rotating conditions was significantly less than it was within the same condition (repeated measures ANOVA: F_2,22_ = 19.4, *p*<0.001, Newman-Keuls post hoc comparison *p*s<0.001).

### Cross-Episode Activation of Ensemble Activity Patterns

**Figure 2:**
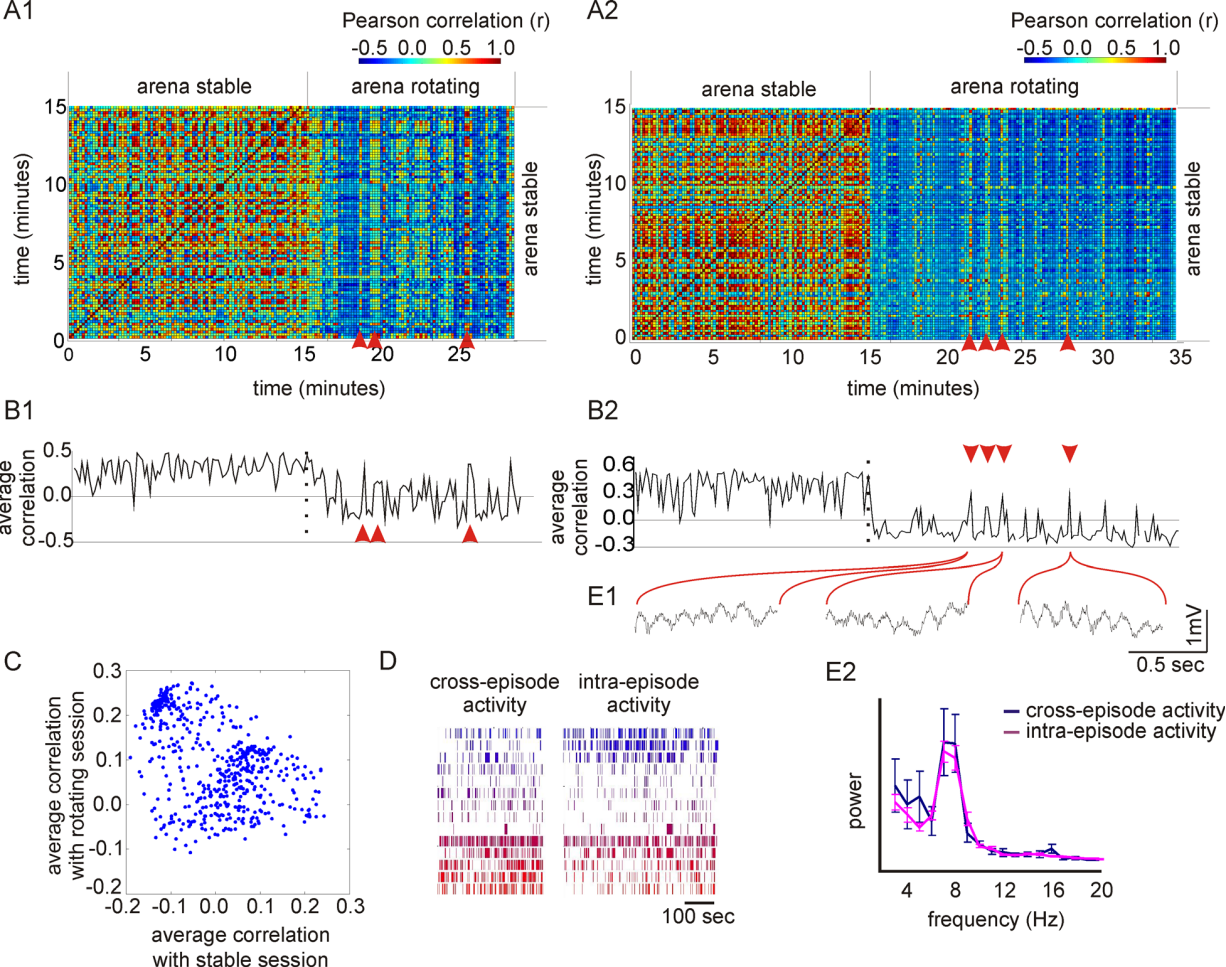
Cross-episode retrieval of ensemble activity. (A) Ensemble activity during different 10-s intervals during the stable and rotating conditions was compared. Two different recordings are shown in panels A1 and A2. Different 10-s intervals during the stable condition tend to have high correlation. Intervals during the rotating condition are often dissimilar to the intervals during the stable condition. However, some 10-s intervals recorded during rotation are highly correlated with activity during the stable condition, as indicated by the reddish stripes (red arrowheads). These are intervals of cross-episode retrieval during which the ensemble activity pattern spontaneously switched to the pattern that was characteristic of the stable condition. (B) Average correlation of each 10-s interval with activity during the stable condition. Intervals during rotation typically have low correlation with activity from the stable condition. However, at intervals of cross-episode retrieval, by definition, the correlation is high, indicating that the ensemble activity pattern was similar to the stable condition. Plots B1 and B2 correspond to the sessions shown in A1 and A2, respectively. (C) The average correlation with activity during the stable condition and the average correlation with activity during rotation, for each 1-s interval of a recording during the rotation condition. The activity organizes into two clusters, representing two ensemble states: cross-episode intervals and intra-episode intervals. (D) Raster plots of activity of the ensemble of 15 cells during the rotating condition, divided into the intervals of cross-episode and intra-episode activity. (E) (E1) Examples of 1-s intervals of hippocampal LFPs from the rotating condition during intervals of cross-episode retrieval of activity that was characteristic of the stable condition. (E2) The average power-spectra of the LFPs during periods of cross-episode activity (blue) and intra-episode activity (magenta). Both activity patterns were expressed during prominent theta oscillations. The analysis of the complete data sets from the recording in plot B2 is shown.

The low but positive (*r* = 0.20±0.07; t_11_ = 2.857; *p*<0.01), average correlation between ensemble vectors during the stable and rotating conditions suggests that hippocampus ensemble activity was not independent during the two conditions. The correlation matrix comparing ensemble activity between all pairs of intervals within and between the stable and rotating conditions shows that during rotation, periods of close-to-zero or negative correlation with activity from the stable condition were intermingled with periods of high correlation with activity from the stable condition as if the hippocampus representation of the stable condition was transiently activated while the rat was in the rotating condition (see red arrowheads in Figure 2A,B). These moments when ensemble activity during rotation was highly correlated to the activity from the stable condition indicate cross-episode retrieval because the hippocampus expressed ensemble activity that was characteristic of the previously experienced stable episode. We refer to these intervals as “cross-episode” in contrast to “intra-episode” intervals during the rotating condition when ensemble activity was characteristic of the rotating condition. The dynamics of the cross-episode retrieval can be visualized by averaging the correlations between ensemble activity vectors at each interval during rotation and all the intervals during the stable condition, which generates a time series of values that characterize similarity of ensemble activity to the stable condition as a function of time (Figure 2B). The distinct intra-episode and cross-episode states of ensemble activity for an exemplar session are illustrated in Figure 2C and 2D. Across all the recordings in the rotating condition, a substantial proportion of the time (27.8%±4.6%) activity was better correlated with the cross-episode state (activity from the stable condition) than it was with the intra-episode state (the activity that was characteristic of the rotating condition), suggesting there were distinct place codes for the global experiences of the stable and rotating conditions.

An analogous analysis of the stable sessions preceding the rotating sessions showed evidence of cross-episode activity—that is, activity during the stable session that was similar to activity during the rotating condition 14.2%±3.8% of the time. The occurrence of cross-episode activity was more frequent during the rotating task than during the stable task (t_11_ = 2.4, *p*<0.05). We also compared the correlation of activity during the rotating task with activity during the stable sessions that preceded and followed the rotating task. The paired *t* test did not indicate a significant difference between the two stable sessions (t_11_ = 0.2158, *p*>0.05). In all subsequent analyses we characterize the cross-episode activation during the rotating task.

Next we characterized changes in firing rates during intra-episode and cross-episode intervals as well as firing rates during the stable condition. The comparisons relied on a similarity measure [15]:
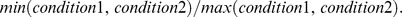
Firing rates for each condition were determined as the total number of action potentials observed, divided by the total recording time; “min” is the smaller of the pair of values and “max” is the larger of the two values. Firing rates during the rotating intra-episode intervals were as distinct from firing rates in the stable condition (0.41±0.03) as were firing rates of randomly paired cells during two sessions in the stable condition (0.40±0.02; t_265_ = 0.16, *p* = 0.87), which is expected for remapping of CA1 ensemble discharge between the stable and rotating conditions [15]. However, during cross-episode reactivation intervals, the firing rates of individual cells were as similar to the rates during the stable session (0.63±0.02) as the firing rates during two sessions in the stable condition (0.63±0.02; t_265_ = 0.06, *p* = 0.95), consistent with cross-episode retrieval of the stable ensemble activity. Thus, according to this metric, activity during the rotating session transiently remapped to resemble the activity that was observed during the stable session, indicating a code for the two task conditions.

**Figure 3:**
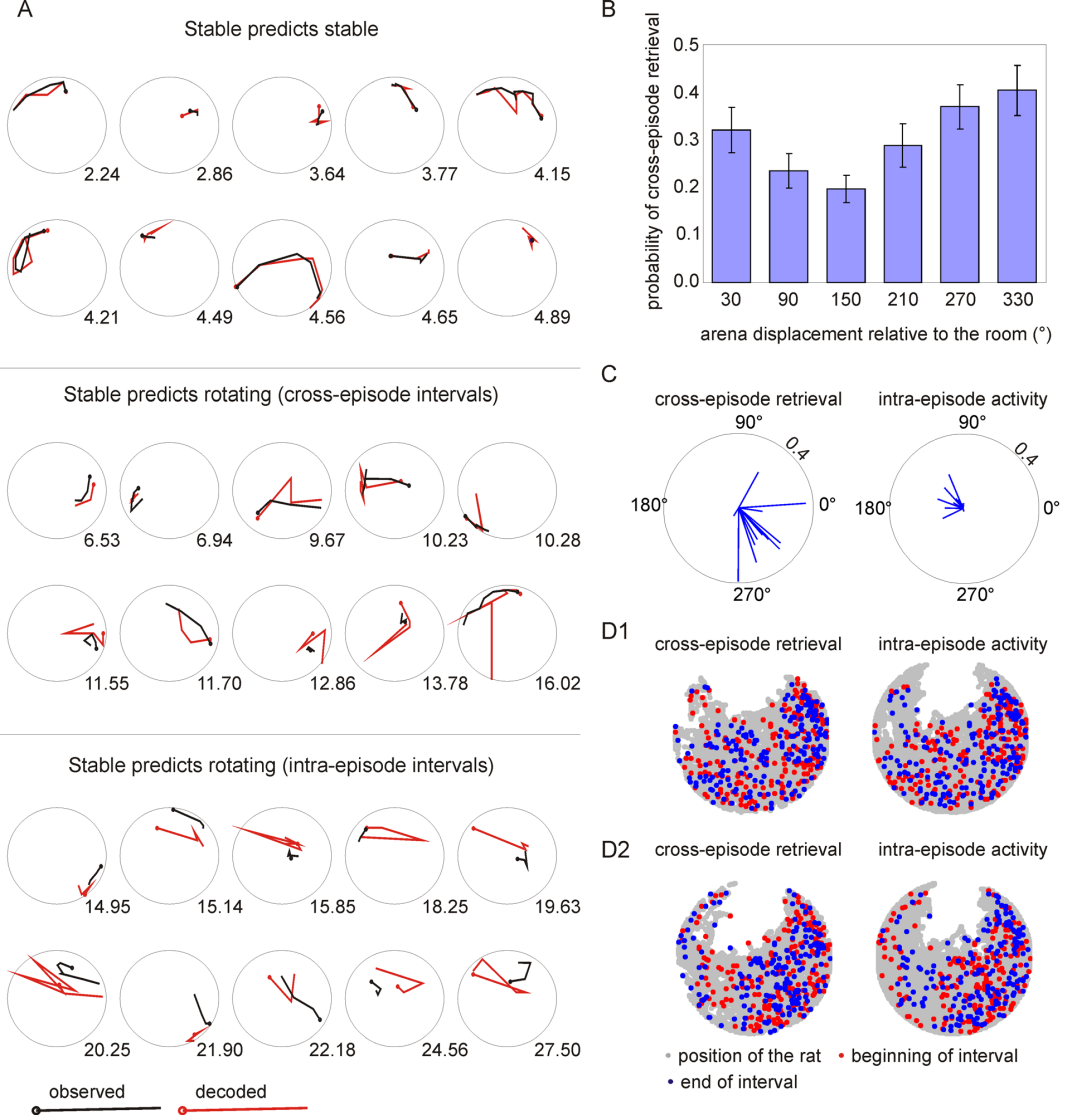
Influence of external cues on cross-episode retrieval. (A) Examples comparing the ability to decode the rat's position during the stable and rotating conditions. The upper 10 plots display the 10 intervals with the best decoding during the stable condition. The middle 10 plots display the 10 cross-episode intervals with the best decoding. The bottom 10 plots display the 10 intra-episode intervals with the best decoding. In all cases the position was decoded using the spatial activity during the stable session as a template. The observed position of the rat is shown in black, the decoded position in red. The number next to each plot indicates the decoding error in cm. (B) The proportion of 1-s cross-episode intervals was computed for different angular displacements of the arena from its orientation in the stable condition. The probability of cross-episode retrieval was highest when the arena displacement was close to 0°—similar to the arena orientation in the stable condition. (C) The average arena displacement vectors are shown for each session during the cross-episode intervals and the intra-episode intervals. The distribution was not random in 11 out of 12 ensemble recording sessions (Raleigh's test, *p*s<0.005); cross-episode retrieval was most likely when the arena displacement within the room was between 270° and 360°. (D) The position of the rat during cross-episode intervals and intra-episode intervals is shown in the spatial frame of the room (D1) and the spatial frame of the arena (D2). Red and blue dots mark the beginnings and ends of the intervals. The data from a single session are shown.

To evaluate whether the cross-episode reactivation might reflect a recollection that is associated with the rat's current location, we used what has been called a “generative model” to examine whether the location-specific activity from the stable condition can be used to decode the rat's position during cross-episode retrieval [11],[16]. The location-specific activity during the stable condition could be used as a reference template to decode the rat's position during rotation better than chance during cross-episode intervals, but not during the intra-episode intervals. Using the location-specific activity from the stable session as a reference template reduced the error in locating the rat during the same stable session to 44.9%±3.2% of the error associated with chance (t_9_ = 17.1, *p*<10^−6^). The same procedure decreased the decoding error during cross-episode intervals to 72.4%±6.5% of chance (t_9_ = 4.3, *p*<0.001), but it did not decrease the decoding error during intra-episode intervals (101.0%±12.0% of chance; t_9_ = 0.1, *p* = 0.46). This tendency was observed both for position in the room and position on the arena (room frame, ANOVA F_2,18_ = 11.2, *p*<0.001; arena frame, ANOVA F_2,18_ = 7.6, *p*<0.005). Thus ensemble activity during cross-episode intervals conveyed information about the rat's current position in the environment (Figure 3A), consistent with a global place code for distinguishing the two task conditions. Indeed, the frequency of the rats making errors (i.e., entering the shock zone) was not significantly higher during cross-episode retrieval (t_11_ = 1.49, *p* = 0.165).

### Hippocampal LFP and Overall Pyramidal Cell Activity Is Similar During the Intra-Episode and Cross-Episode States

We then asked whether the intra- and cross-episode activity differences could have resulted from distinct states of overall neural activity, local field potential (LFP) oscillations, or behavior. The average firing rate of the ensemble did not differ between intra- and cross-episode intervals in any of the 12 recordings we analyzed (*t* tests, all *p*s>0.05), suggesting levels of network activity were the same. Next we compared LFPs from the hippocampal pyramidal cell layer during the intra- and cross-episode activity from the 11 experiments where LFPs were recorded. We focused on 4–10 Hz theta oscillations (Figure 2E), which are associated with locomotion [17]. Two-way ANOVAs comparing the effects of oscillation frequency and the type of interval confirmed a significant effect of frequency in all 11 recording sessions, showing significant theta modulation. In contrast, there were no significant effects of interval type or the interaction (*p*s>0.05 with Bonferroni corrections for 11 comparisons), indicating that the power of theta oscillations was not different between the intra- and cross-episode states. Similarly, the rat's speed was not different during the intra- and cross-episode intervals (*p*s>0.05 with Bonferroni corrections for 12 comparisons).

We could find no evidence to suggest that the differences in the ensemble activity patterns during the intra- and cross-episode intervals are a consequence of distinct behavioral or functional hippocampal states.

### Characterizing Transitions Between Intra- and Cross-Episode Activity

We next investigated the transitions between the intra- and cross-episode activity states. We analyzed the time series of differences between the average correlation with the stable condition and the average correlation with the rotating condition. Moments of time that were preceded by at least 3 s of lower than median values and followed by at least 3 s of higher than median values (and vice versa) were detected as transitions. During the 2-s period around the time of a transition the rats moved faster (15.2±1.0 cm/s) than the session-averaged speed (11.7±0.7 cm/s; paired *t* test: t_11_ = 3.48, *p* = 0.005), indicating the rats were relatively active during the transitions to and from cross-episode retrieval of the discharge patterns, instead of being relatively immobile as has been reported for other forms of memory-associated replay of hippocampus place cell discharge [7],[18]–[20]. Similarly, single unit firing rates during transitions (1.04±0.1 AP/s) were also greater than during randomly chosen intervals (0.78±0.1 AP/s; t_11_ = 2.96, *p* = 0.013). In contrast, differences in the power of theta oscillations or the coordination of firing amongst cells were not detected during transitions compared to randomly chosen intervals (unpublished data), providing further evidence that the cross-episode retrieval of the discharge patterns is behaviorally, electrophysiologically, and probably also functionally different from the events that are typically associated with immobility and sharp wave associated ripples (SWRs) in the LFP that have been previously reported [7],[18]–[20].

### Environmental Cues Influence Cross-Episode Retrieval

Retrieval cues influence what details of an episode people recollect, so we tested whether the occurrence of cross-episode activity was influenced by the details in the arrangement of environmental cues, which were identical across the stable and rotating conditions except for the arena rotation. During the rotating condition, once a minute the angular position of the arena in the room was the same as it was in the stable condition. To investigate if the environmental similarity influenced cross-episode retrieval of discharge patterns, we investigated the relationship between ensemble activity and the angular position of the arena as it rotated. Each recording session was divided into six categories, each category corresponding to a 60° range of the arena displacement. Cross-episode retrieval of discharge patterns occurred at all arena displacements, but it was most probable when the clock-wise rotating arena was displaced between 270° and 360°, which is when the arena was approaching the same orientation as in the stable condition (Figure 3B).

To assess the significance of the effect, the distribution of angular displacement vectors during all cross-episode events was determined for each of the 12 recordings separately, and the Rayleigh test was performed for each recording to test whether the distribution of arena displacements is random. The mean vector was computed and the magnitude (length) of the mean vector and the number of cross-episode events were used to determine the z value. In one of the 12 recordings, the effect of arena displacement on cross-episode retrieval was not significant. In the remaining 11 recordings, the effect was significant with *p* values smaller than 0.005 in each of the 11 cases. Importantly, the directions of the mean vectors for the 12 recordings indicated that the cross-episode retrieval events were most probable when the arena was displaced between 270° and 360° as shown in Figure 3C (left panel). The significance of this effect was confirmed by a separate Rayleigh test in which the distribution of the mean vectors of the cross-episode retrieval events for the 12 recording session was analyzed (Z_11_ = 6.59, *p*<0.001). Thus, dynamic, physical properties of the environment influenced what otherwise appears to be spontaneous cross-episode retrieval of hippocampal spatial representations (Figure 3D).

### Influence of the Information in Hippocampal Discharge Just Prior to Cross-Episode Retrieval

**Figure 4:**
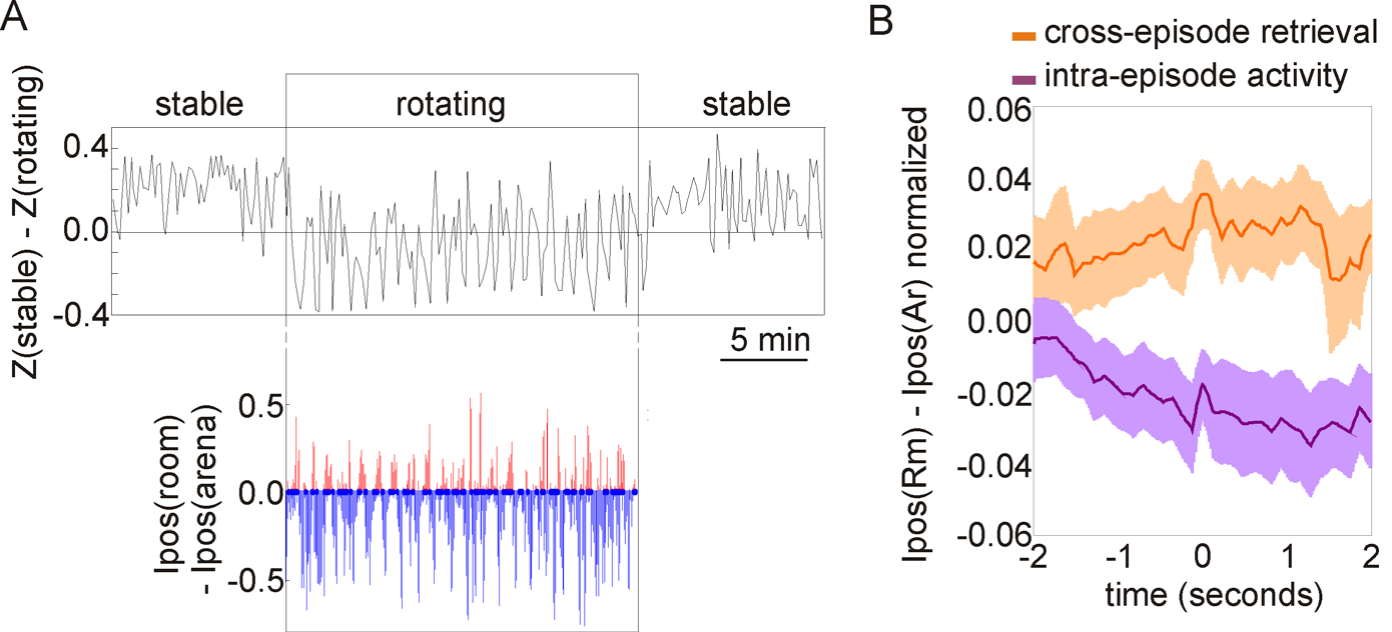
The probability of cross-episode retrieval is influenced by ongoing hippocampal ensemble activity. (A) The temporal dynamics of the code for task variant and code for spatial frame during a single rotating session flanked by stable sessions. (Upper plot) The code for task variant. A time series measuring the similarity (r transformed to z scores) of ongoing ensemble activity to activity during the stable and rotating conditions. Cross-episode retrieval is apparent as deflections during rotation that are similar to the values during the stable condition. (Lower plot) The code for spatial frame. A time series measuring the preference in ongoing ensemble activity for representing information about the current position in the room or arena. Each few seconds, positional information toggles between preferentially representing positions in the room (red) or arena (blue) spatial frame. (B) Averaged normalized spatial frame preference during rotating condition intervals of cross-episode retrieval and intra-episode activity. Time 0 corresponds to the moment when cross-episode activity was detected (orange) and the time when intra-episode activity was detected (purple). The analysis shows that cross-episode retrieval is preferentially associated with information about position in the room, whereas the intra-episode activity during rotation is preferentially associated with information about position on the arena.

To investigate whether the information “in mind” influences cross-episode retrieval as it does in people, we exploited our earlier finding that during the place avoidance task in the rotating condition, the positional information in hippocampal discharge alternates between the rotating and stationary spatial frames [13]. Momentary positional information (*I_pos_*) was used to estimate the information that ensemble discharge is providing about the rat's position in the stationary room and the rat's position on the rotating arena. At each moment, ensemble discharge preferentially signals position in one of the two spatial frames [13]. We thus asked whether the information about the spatial frame had an influence on cross-episode retrieval (Figure 4).

Cross-episode retrieval was more likely to occur when the stationary room frame locations were preferentially represented. The 20% of intervals with the highest preference for room information were not only predominantly associated with cross-episode activity, but these intervals also tended to precede cross-episode retrieval. The 20% of intervals with the highest preference for arena information were associated with intra-episode activity (Figure 4B). Consistent with the notion that internal state influences episodic recall, the likelihood of cross-episode retrieval was related to the type of information (about spatial frame) that was currently being expressed in the hippocampal discharge.

### Concurrent Expression of Different Categories of Information Within Ensemble Discharge

Combining different categories of information into a single perceived experience is a hallmark of episodic memory. Here we observed and characterized two types of information in hippocampal discharge during the place avoidance task in the rotating condition. In prior work, we showed that information about location in the spatial frame of room and information about location in the spatial frame of the arena are represented and dynamically organized in hippocampal discharge [13]. In this article we characterize the dynamic organization of the information about one of two distinct variants of the place avoidance task that the rat knows. This provides an opportunity to study the dynamic interactions amongst ensemble representations of these two categories of information, to our knowledge for the first time (Figure 4A).

**Figure 5:**
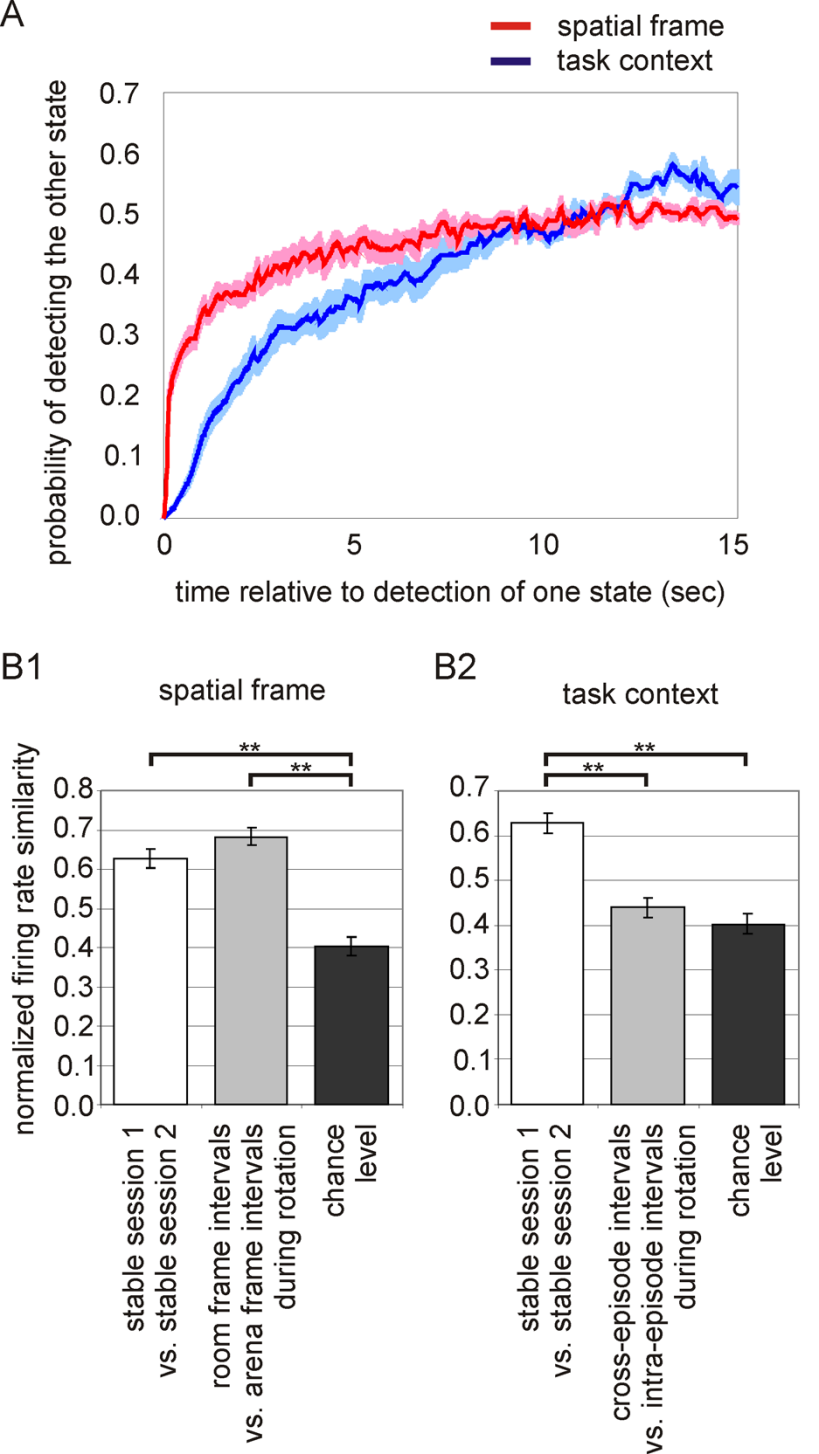
Organization of hippocampal ensemble activity according to two distinct categories of spatial information. (A) Cross-correlation plots indicating probability of the code for spatial frame-specific position (red) and the code for task variant (blue) to switch to representing the other value. The code for spatial frame: Time 0 marks that activity was preferentially signaling position in either the room or the rotating arena. The *y*-axis shows the probability of preferentially signaling position in the other frame within the time interval indicated on the *x*-axis. The probability of observing activity that represents position in the other frame remains low for approximately 7 s when it reaches a plateau. The code for task variant: Time 0 is the time that cross-episode or intra-episode activity was preferentially expressed, and the *y*-axis shows the probability of the other activity pattern being expressed. The probability of observing the other pattern remains low for about twice as long as is the case for the spatial frame code, before reaching a plateau. Thus the code for task variant predicts activity further into the future than the code for spatial frame. (B1) The firing rate similarity between room frame intervals and arena frame intervals was high. The similarity was indistinguishable from the similarity of firing rates during two stable sessions in the same environment, and it was greater than is expected by chance. (B2) The firing rate similarity between cross-episode intervals and intra-episode intervals was low. The similarity was not higher than would be expected by chance.

First, we compared the dynamics of ensemble changes in the information about spatial frame and information about task variant. For both types of information we created a plot that shows, for each time interval, the probability of observing the opposite ensemble state than the state observed at time zero (Figure 5A). In the category of spatial frame, the probability of observing discharge associated with positions in the other spatial frame remains low for approximately 7 s, then it reaches a plateau (Figure 5A), consistent with our prior report [13]. In the category of task variant, the current state is likely to persist substantially longer than the persistence of information about spatial frame (Figure 5A). Next we analyzed the firing rate changes of cells in distinct ensemble states. For both categories of information, we compared the firing rate of cells between the periods of time in the two ensemble states for each category, using the firing rate similarity measure (Eq. 1; Figure 5B). The average firing rate similarity between the periods of time that are dominated by frame-specific, arena, or room position information (|I_pos_|>0.2) is indistinguishable from the firing rate similarity between two sessions in the same environment, and greater than would be expected by chance (F_2,246_ = 42.6, *p*<0.0001, Newman-Keuls *p*<0.001, Figure 5B1). In contrast, the average firing rate similarity between the cross-episode and intra-episode periods is low, not higher than what would be expected by chance (F_2,246_ = 25.9, *p*<0.0001, Newman-Keuls *p*<0.001, Figure 5B2). This shows that the representations of the two distinct task variants are more different from each other than the set of representations of position in the two distinct spatial frames. Compared to the category of spatial frame, the category of task variant changes slower and is characterized by larger firing rate changes in ensemble activity. These differences suggest that the two types of information are controlled by bona fide distinct mechanisms of neural activity.

## Discussion

### Cross-Episode Retrieval of Neuronal Activity

During dynamic steady-state behavioral conditions, we observed two distinct codes in hippocampal ensemble discharge. One code signaled a distinction between the current, rotating task variant and the stable task variant from a prior behavioral episode. The other was a place code that signaled the spatial frame of the rat's current position as the arena rotated. The characteristics and interactions between the two types of information confirmed three predictions from the theory of (re)constructive episodic memory recollection. (1) A commingling of neural activity from distinct episodes: At the level of task variant, we identified a cross-episode retrieval of hippocampal ensemble discharge patterns during active behavior, demonstrating a comingling of hippocampal neural representations of distinct behavioral episodes during dynamic steady-state environmental conditions (Figures 1 and 2). (2) We found an influence of environmental/retrieval cues on the expression of the memory traces: We observed that the cross-episode retrieval occurred when the rat was at seemingly random locations, but was more likely when the arena was returning to the same orientation that it occupied in the stable condition (Figure 3). (3) The interaction between the two types of information revealed an influence of the subject's internal state on memory retrieval: The cross-episode retrieval of the stable condition was more likely when the place code for spatial frame represented stationary locations better than rotating locations (Figure 4). These observations demonstrate properties of ecphoric recollection of episodic memory in hippocampal discharge of the rat [5].

The present findings in rats have impressive similarities to reports from human subjects that patterns of field potentials in the electrocorticogram are reactivated during the free recall of word and picture lists. In particular, during recall of a specific item, the neural activity correlates of other items were more likely the closer those items were to other semantically related study items [21],[22], which resembles our observation that cross-episode neural activity representing the stable condition was most probable during the rotating condition when the arena was returning to its orientation during the stable condition (Figure 3). Furthermore, patterns of human field potentials that are associated with specific study items were more probable if the subject was recollecting a categorically related study item [23], which is rather similar to our observation that the cross-episode neural representation of the stable condition was more likely during the rotation condition when the hippocampal place code preferentially signaled room frame positions (Figure 4). These dynamic features of free memory recall are called “contiguity effects” and are taken as evidence for the organization of episodic memories and mental time travel in human subjects [24]. It is remarkable that similar organizational features in the physiological expression of memory are observed in the hippocampal neural discharge of the freely behaving rat.

Our findings differ from those reported in a number of studies that investigated the reproduction (replay) of rat hippocampal discharge patterns that characterized a prior experience. Those studies were conducted under the hypothesis that replay is part of the process of consolidating recent experience into long-term memory [25],[26]. Indeed, replay was observed in sleeping rats [27]–[31] as well as in awake rats during SWRs, which are typically associated with relative immobility [7],[8],[18],[32]–[34] but that can also be observed during active exploration [20]. In contrast to prior work, the present study was motivated by the (re)constructive memory framework, which is why we investigated interactions between the reactivated ensemble discharge patterns associated with a distinct behavioral episode, the activity patterns associated with the current episode, the environmental retrieval cues, and other distinct types of information that were being processed in hippocampal activity just prior to and during the retrieval. We found that cross-episode reactivations occurred during sped-up locomotion, when the hippocampal LFP was dominated by theta oscillations (Figure 2E), rather than SWRs. In contrast, prior work that reported discharge patterns from prior experience in distinct environments [18] and the expression of novel sequences of place cell discharge that were associated with the present environment [33] observed these phenomena during the SWRs associated with relative immobility. In this respect our results are analogous to those of Jezek and colleagues [12], who observed a flickering of hippocampal representations at the time of transitions between two spatial contexts in rats during active exploration associated with hippocampal theta activity. Our work complements and extends these findings by showing that the cross-episode reactivation is not limited to moments of experimentally induced “teleportation” between two conditions but that it occurs throughout an experiment in dynamic steady-state conditions. When taken together, the separate observations of cross-episode retrieval during active exploration and SWR-associated replay of discharge patterns offer a potential mechanism for how novel associations and representations of information that were never directly experienced might be created and then stored in long-term memory. This offers an explanation for how both new knowledge and false memories can be created in the mammalian brain without the information being a direct record of experience.

### Distinct Dynamic Processes Organize Hippocampal Discharge According to Two Categories of Information

We identified in prior work that information from the spatial frame of the room and the spatial frame of the arena was preferentially processed at distinct times while the rat performed the two-frame place avoidance task [13]. The present findings build on this, demonstrating that the discharge of the same dorsal hippocampal principal cells is organized in time into functionally distinct coactive groups according to at least two categories of information during steady-state conditions (Figure 5). One category represents the task variant and the other represents spatial frame-specific position. Combining different aspects (or categories) of experience into a single coherent experience is a characteristic of episodic memory and these data may offer an initial glance into the neural mechanisms underlying the process.

Two types of spatial information have been previously described and characterized in hippocampal discharge [35]. One is global information about the context in the sense of the spatial environment [36] or task [37]. This information is signaled by the subset of hippocampal pyramidal cells that are active in a particular context. The other type of information is more local and encodes locations within a particular environment signaled by location-specific changes in the firing rate of place cells [38]. In this work we characterized the ensemble signal about the task, which corresponds to the global code for context, and we characterized the signal for the rat's position in the room and arena frame, which corresponds to a more local spatial signal. In our experimental situation we could compare the two types of signal directly within the same paradigm. Distinct properties were associated with the dynamic functional grouping that organized hippocampal discharge into the representations of local (position) and global (task variant) categories of spatial information. Only small changes in firing rates were associated with the relatively rapid switching between representing local positions in the two spatial frames (Figure 5A), whereas the firing rates changed radically (remapped) when the representation switched between representing the different task variants and these changes persisted substantially longer (Figure 5B). These observations are consistent with the idea that hippocampus represents different categories of information by dynamically organizing coactive neurons into same-function groups on different timescales [39].

We also found that the two categories of spatial information were processed conjointly because the local and global place codes were combined in a nonrandom manner. For example, representations of the stable task condition in the global place code were preferentially associated with positional information from the stationary spatial frame in the local place code. This is consistent with a canonical feature of episodic memory recollection, in which different categories of experience (objects, places, events, etc.) combine into coherent episodes of experience, but the combinations are not random. Some places are more likely to be associated with particular objects and events, and other places with different activities. The data presented here offer a first glimpse and a paradigm for studying how representations of different categories of information combine and interact within ensemble discharge.

Accumulating evidence from this and other work [9]–[11],[13],[40] suggests that hippocampal activity is less homogeneous and more variable than was traditionally believed, and by averaging the place cell discharge over the entire session one is prone to miss fundamental features of hippocampal information processing [16]. We wonder whether at least some of the reports of rate remapping [35] could be partially accounted for by the representation being present only during part of a recording session. As shown here, signature features of episodic memory processing and dynamical functional grouping to process different categories of information in familiar, steady-state conditions can be decoded from hippocampal ensemble discharge by analyses that do not assume stationarity of the spike trains. We have been especially impressed to observe such clear, unambiguous signs of higher cognition in the rat.

## Materials and Methods

### Ethics Statement

The experimental and analytical procedures were previously published in detail [13]. The procedures complied with NIH and institutional guidelines and were approved by Downstate Medical Center's Institutional Animal Care and Use Committee.

### Place Avoidance Task Variants

Seven adult male Long-Evans rats (Taconic Farms, DE) were used. The same rats were trained in two variants of the active place avoidance task in the same environment (Figure 1A and 1B). In both task variants, rats had to avoid the location of a mild foot shock (constant current, 60 Hz, 0.3 mA, 500 ms) while foraging for food pellets that were scattered on a circular arena (82 cm diameter) from an overhead computer-controlled feeder. The shock zones were 45° wide and spanned the outer 60% of the arena diameter. In one task variant the arena was *stable* and the rat had to avoid a single shock zone. In another task variant, the arena was *rotating* (1 rpm) and the rat had to avoid two shock zones: one shock zone was defined by the stationary room cues, and the other defined by rotating arena cues [13],[41]. The rat was food deprived and reinforced to walk on the arena and collect food pellets that were periodically (every 20 s) dropped to random locations on the arena surface from the overhead feeder. The position of the rat and angular displacement of the arena in the room was monitored by an overhead camera. The cable connected to the rat's back was used to deliver foot-shocks, to supply power for the LED on the rat used to track the rat's position, and to transmit the electrophysiological signal from electrodes implanted in the rat's hippocampus. The electrophysiological recordings were made across a sequence of three tasks, stable–rotating–stable. The electrophysiological recordings were made in steady-state conditions after the rats were very familiar with the tasks (>10 sessions).

### Electrophysiology

Ensembles of single complex-spike cells were recorded from the dorsal CA1 region of the hippocampus while rats performed the two place avoidance tasks. The recordings were done using position tracking methods, tetrode-configured electrodes, and electrophysiological recording and analysis techniques that have been described in detail [13]. We recorded 224 well-isolated neurons, 215 complex spike cells (putative pyramidal cells), and nine theta cells (putative interneurons). For the ensemble analysis, we used 12 recordings that contained 10 or more complex spike cells during the stable–rotating–stable recording sequence. Ensemble discharge was analyzed to decode two kinds of information: (1) information about task variant represented by ensemble discharge and (2) information in ensemble discharge about the rat's separate positions in the spatial frames of the stationary room and the rotating arena.

### Assessing the Ensemble Representations of the Place Avoidance Task Variants

During short time intervals, we decoded whether the ensemble activity represented the stable or the rotating condition. The ensemble activity during a fixed time interval (e.g., 1 s) was characterized by the ensemble spike-count vector that describes the number of action potentials that each cell emitted during the time interval (Figure 1C). The similarity of ensemble discharge during two time intervals was quantified by computing the Pearson correlation of the two vectors. A correlation matrix was constructed to organize all the pair-wise comparisons between the set of spike-count vectors in a session. The matrix was used to visualize the change in ensemble discharge patterns between and within the stable and rotating tasks (Figure 1D). The Pearson correlations are reported in the manuscript, but they were z-transformed for statistical comparisons.

### Assessing the Information About the Position in the Room Frame and Arena Frame Represented in Ensemble Discharge

An important feature of the place avoidance paradigm is that during the rotation condition, CA1 ensemble discharge is multistable, toggling between representing the rat's location in the spatial frame of the stationary room and representing the rat's location in the spatial frame of the rotating arena [13]. We used this signal to determine whether cross-episode retrieval of the stable representation during the rotation task is influenced by the current “state of mind,” specifically the processing of information about frame-specific position in hippocampal discharge just prior to cross-episode retrieval. It was thus necessary to decode which spatial frame was being represented by location-specific CA1 discharge. We computed ensemble *I_pos_* as described in detail and used for the same purpose in prior work [13]. *I_pos_* estimates the information about the rat's position in one spatial frame during a short time interval, 117 ms in this case. First *I_pos_(t)* is computed on the basis of the discharge of a single cell at each moment *t* for the rat's position in the room (stationary) frame and separately for the rat's position in the arena (rotating) frame. Then to compute ensemble *I_pos_(t)*, the frame-specific sum of the *I_pos_(t)* values at each moment is calculated. The difference between the room frame *I_pos_(t)* and arena frame *I_pos_(t)* is computed to determine the momentary frame preference in the ensemble discharge for a given time interval.

### Position Reconstruction

The ensemble activity vector at each time step was used to reconstruct the rat's path using a straightforward template-matching method [9],[11],[42]. At each location, the firing rate of each cell in the ensemble was used to construct a location-specific template firing rate vector. The predicted current location was the position that maximized the projection of the current firing rate vector onto one of the location-specific template vectors. If there was no activity during a time step, no prediction was made. Only recordings with at least 10 active cells (>0.1 spikes per second) during both the stable session and rotating session were used for position reconstruction (10 sessions). This method explicitly tests how well location-specific firing rate itself predicts the current location [9],[42],[43]. The prediction at each time step is independent of the activity and prediction at other time steps. The average distance between the reconstructed and observed locations (in pixels) was used to evaluate the accuracy of the reconstruction.

## Abbreviations

LFP: local field potential
SWR: sharp wave associated ripple
